# IL-22 activates the PI3K-AKT pathway to promote colorectal cancer cell proliferation and metastasis

**DOI:** 10.1007/s12672-024-01169-9

**Published:** 2024-07-29

**Authors:** Hong-xun Ruan, Xiao-ning Qin, Wei Huang, Lin Lin

**Affiliations:** Department of General Surgery III, The Second Hospital of Hebei Medical University, 215 Heping West Road, Shijiazhuang, 050000 Hebei China

**Keywords:** Colorectal cancer, Interleukin-22, PI3K/AKT signaling pathway, Proliferation, Metastasis

## Abstract

**Background:**

Colorectal cancer (CRC) is a prevalent malignancy with high morbidity and mortality rates. Previous studies have demonstrated that interleukin (IL)-22 is involved in CRC progression; however, the exact mechanism remains unclear. This study aimed to investigate the effects of IL-22 on CRC cell proliferation and metastasis.

**Methods:**

IL-22 levels in the serum and tissues of CRC patients were measured using enzyme-linked immunosorbent assay (ELISA). Cell counting kit-8 (CCK-8) assay was used to detect the viability of CRC (HCT116) cells treated with different IL-22 concentrations. Colony formation, Transwell invasion, and scratch assays were employed to assess the effects of IL-22 on cell proliferation, invasion, and migration. Western blotting was performed to measure the expression levels of phosphatidylinositol 3-kinase (PI3K), protein kinase B (AKT), p-PI3K, p-AKT, E-cadherin, matrix metalloproteinase (MMP)-2, MMP-9, SNAI1, and TWIST1 in HCT116 cells treated with IL-22 or a PI3K inhibitor.

**Results:**

ELISA results showed that the expression of IL-22 was significantly increased in the serum and tissues of CRC patients compared to controls. IL-22 treatment increased cell viability and colony formation in a concentration-dependent manner and enhanced cell invasion and migration. Western blotting analysis revealed that IL-22 stimulation upregulated p-PI3K and p-AKT expression, while total PI3K and AKT levels remained unchanged. Additionally, IL-22 also decreased E-cadherin expression and increased the expression of MMP-2, MMP-9, SNAI1, and TWIST1.

**Conclusions:**

IL-22 activates the PI3K-AKT pathway and promotes HCT116 cell proliferation and metastasis. Targeting the regulation of the PI3K/AKT pathway may be a potential therapeutic strategy for CRC.

**Supplementary Information:**

The online version contains supplementary material available at 10.1007/s12672-024-01169-9.

## Introduction

Colorectal cancer (CRC) is a common malignancy of the digestive system with high morbidity and mortality worldwide [1]. Despite significant improvements in CRC treatment over recent decades, the pathogenesis of this disease remains incompletely understood. Epidemiological studies have highlighted the importance of dietary and lifestyle in CRC risk [2, 3]. A high-fiber diet, ample intake of vegetables and fruits, and limited consumption of processed meat products can significantly reduce CRC risk [2, 3]. Genetic factors also play a crucial role in CRC development, with certain mutations significantly associated with increased susceptibility [4, 5]. Understanding the mechanisms behind these factors is essential for identifying new targets for CRC prevention, early diagnosis, and treatment.

Interleukin (IL)-22, a cytokine involved in immune responses, has been found at elevated levels in various tumors, including gastric, lung, and breast cancers [6–8]. In CRC, IL-22 has been implicated in tumor proliferation and metastasis [9].Giannou et al. demonstrated that IL-22 binding protein (IL-22BP) regulates liver metastasis progression in CRC using two in vivo mouse models, but it does not affect colorectal lung metastases [10]. Immune cell-derived IL-22 contributes to malignancies by promoting tissue regeneration and repair. Liver and lung metastases of CRC can be prevented in IL-22-deficient and IL-22 antibody-treated mice, whereas IL-22 overexpression promotes metastases [6]. These findings suggest that IL-22 may be a promising therapeutic target in metastatic CRC. However, the precise role and mechanism of IL-22 in CRC remain unclear.

The phosphatidylinositol 3-kinase (PI3K)/protein kinase B (AKT) signaling pathway is critical for cell cycle regulation, proliferation, apoptosis, and tumor metastasis. PI3K activation leads to AKT phosphorylation and subsequent activation of downstream signaling pathways [11–14]. Hyperactivation of the PI3K/AKT pathway has been linked to increased tumor cell proliferation and invasion, as well as reduced apoptosis [15]. In addition, activation of this pathway is associated with downregulation of E-cadherin expression and upregulation of matrix metalloproteinases (MMPs), which are key hallmarks of tumor metastasis [16]. Despite these insights, the interaction between IL-22 and the PI3K/AKT pathway in CRC cells has not been thoroughly investigated.

Therefore, this study aimed to investigate the effects of IL-22 on CRC cell proliferation and metastasis through PI3K/AKT pathway. By elucidating the regulatory mechanisms of IL-22 in CRC development, we hope to provide a new theoretical basis for understanding CRC pathogenesis and to offer guidance for the development of new therapeutic strategies for CRC treatment.

## Materials and methods

### Clinical sample collection

Plasma was collected from CRC patients (*n* = 66) and normal subjects (*n* = 36), and tumor and adjacent noncancerous tissues were obtained from CRC patients (*n* = 66) at The Second Hospital of Hebei Medical University between January 2023 and June 2023. This study was reviewed by the Ethics Committee of the Second Hospital of Hebei Medical University (2022-R754), and all patients signed an informed consent form. The clinical information of patients was shown in Supplementary Table 1.

### Cell culture and grouping

The CRC cell line HCT116 was purchased from the American Type Culture Collection (ATCC) and cultured in Dulbecco’s modified Eagle’s medium (DMEM) supplemented with 10% fetal bovine serum (FBS) and 1% penicillin-streptomycin. The samples were maintained in a 5% CO_2_ incubator at 37 °C for optimal growth.

To observe the effects of IL-22 on HCT116 cells, they were treated with varying concentrations of IL-22 (0, 5, 10, 50, and 100 ng/mL). For further experiments, cells were divided into three groups: control, IL-22 (treated with 100 ng/mL IL-22 for 48 h), and IL-22 + LY294002 (treated with 20 µM LY294002, a PI3K inhibitor, for 30 min, followed by 100 ng/mL IL-22 for 48 h).

### Enzyme-linked immunosorbent assay of IL-22 levels in plasma and tissues

Enzyme-linked immunosorbent assay (ELISA) was conducted to measure IL-22 levels in the serum of CRC patients and normal participants (control group), as well as in CRC and adjacent tissues. Serum and tissue samples were first collected. Serum samples were centrifuged to obtain clear supernatant, while tissue samples were homogenized and then centrifuged to obtain tissue supernatant. Subsequently, the IL-22 concentration was determined using the corresponding ELISA kit according to manufacturer’s instructions. Finally, the absorbance was measured using a multimode microplate reader, and absorbance values were converted to IL-22 concentrations.

### Cell counting Kit-8assay of cell viability

Cells were inoculated into 96-well plates with 5 × 10^3^ cells per well, with 100 µL of culture medium per well. According to the experimental design, various concentrations of IL-22 (0, 5, 10, 50, and 100 ng/mL) were added to the respective wells. After incubation, the cells were processed according to the instructions of the Cell Counting Kit-8 (CCK-8) reagent. A 10 µL of CCK-8 reagent was added to each well. After incubation for 2 h, the absorbance at 450 nm was measured using a microplate reader.

### Colony formation assay

HCT116 cells were seeded and cultured in 6-well plates (500 cells/well) for 2 weeks. The culture medium was discarded, and cells were fixed in 4% paraformaldehyde (Sigma-Aldrich, USA) for 20 min at room temperature. After washing with phosphate-buffered saline (PBS), cells were stained with 0.1% crystal violet solution (Sigma-Aldrich) for 20 min. The number of colonies (clusters of at least 50 cells) was counted using an inverted microscope (×100; Olympus, Japan). Data processing was performed using ImageJ version 1.52.

### Scratch assay to determine cell migration

HCT116 cells were inoculated into 6-well plates (5 × 10^5^ cells/well) and cultured until they reached 90% confluence. Scratches were made with a sterile 10 µL pipette tip, and cell debris was washed with PBS. The wells were replenished with DMEM supplemented with 2% FBS. Finally, cell migration was observed in four randomly selected fields under an inverted microscope (×100; CKX53 IPC; Olympus) at 0 and 24 h and photographed. Migration distance was quantitatively evaluated using ImageJ.

### Transwell invasion assay

Transwell assays were performed to detect HCT116 cell migration. After washing off the residual cell culture medium with PBS, cells were starved overnight in DMEM. HCT116 cells were digested with trypsin and resuspended in serum-free DMEM to adjust the cell density to 1 × 10^6^ cells/mL. The suspension (200 µL) was inoculated into the top chamber and pre-coated with Matrigel, and 800 µL DMEM containing 10% FBS was added to the bottom chamber. The Transwell insert was removed after 24 h incubation in a 5% CO_2_ incubator at 37 °C. Cells in the top chamber were gently wiped with cotton balls. Invasive cells on the bottom side of the insert membrane were fixed with 4% paraformaldehyde for 15 min, rinsed thrice with PBS, stained with 0.1% crystal violet for 15 min, and washed thrice with PBS. Finally, the invasive cells were imaged using an inverted microscope (×40 and ×100; CKX53 IPC; Olympus, Japan) and counted using ImageJ software.

### Western blotting

Radioimmunoprecipitation assay reagent (R0278, Sigma-Aldrich, USA) was used to extract cellular proteins. After centrifugation to remove cellular debris, the supernatant was collected, and the protein concentration was determined using a BCA kit. Equal amounts of protein samples (20 µg) were subjected to sodium dodecyl sulfate-polyacrylamide gel electrophoresis and transferred onto polyvinylidene fluoride membranes. The membranes were blocked with 5% nonfat dry milk at room temperature to prevent nonspecific binding. Specific primary antibodies against PI3K (1:1000), p-PI3K (1:1000), AKT (1:1500), p-AKT (1:1000), E-cadherin (1:1000), MMP-2 (1:2000), MMP-9 (1:2000), SNAI1 (1:2000) and TWIST1 (1:2000), and β-actin (1:5000), which were obtained from Abcam (UK), were added and incubated overnight at 4 °C. Unbound primary antibodies were removed by washing with PBS-Tween 20. HRP-labeled anti-rabbit secondary antibody (Abcam, UK) was added, incubated for 2 h at room temperature, and washed with PBS-Tween 20. An enhanced chemiluminescent reagent was added to the membranes, and the protein bands were acquired using a chemiluminescence apparatus. Grayscale measurements were performed using ImageJ software.

### Statistical analysis

Statistical analysis was performed using SPSS version 26.0. Measurement data conforming to a normal distribution were expressed as the mean ± standard deviation. A *t*-test was adopted to compare two groups, and one-way analysis of variance was used to compare multiple groups (*P* < 0.05).

## Results

### Elevated IL-22 levels in the serum and tumor tissues of CRC patients

**Fig. 1:**
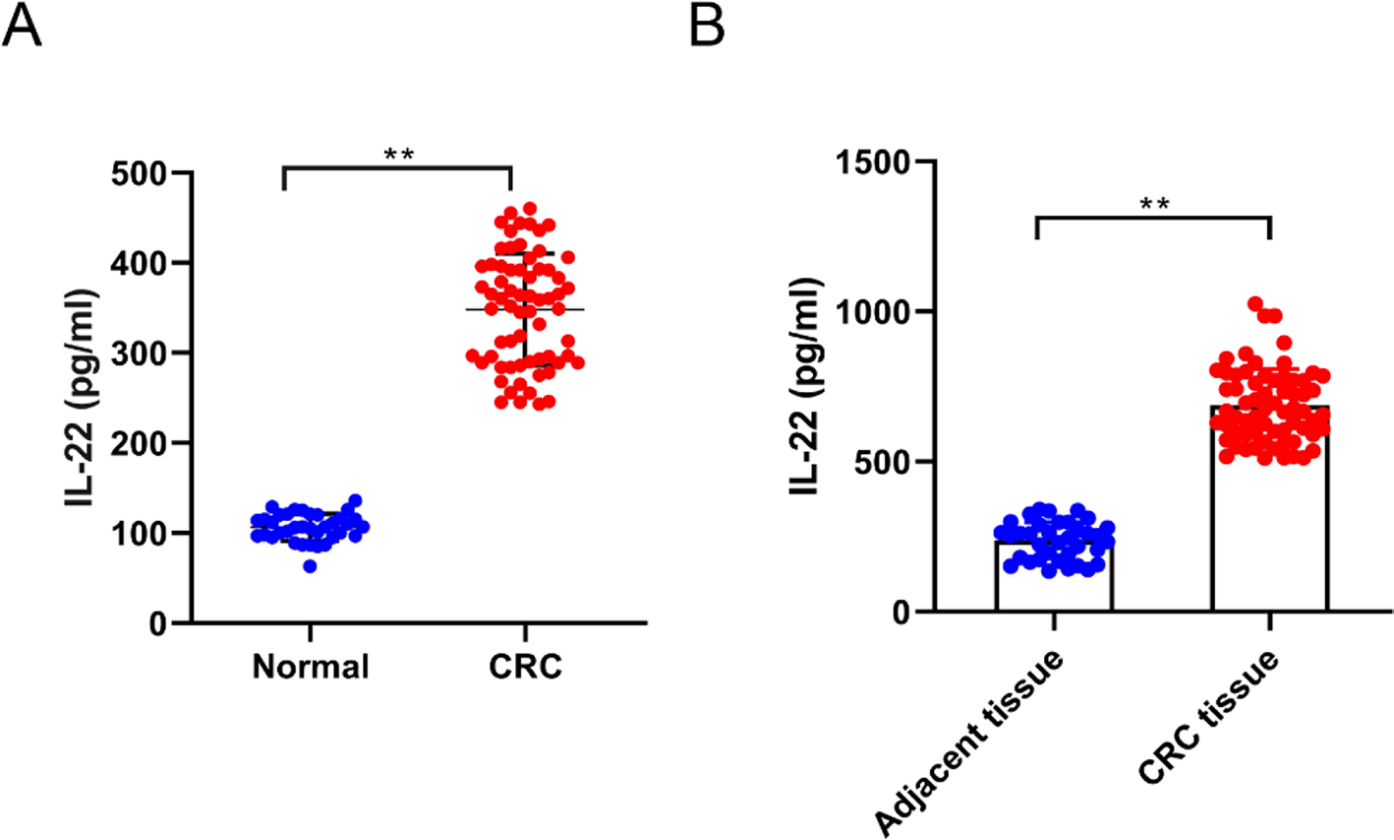
Elevated IL-22 levels in serum and tumor tissues of CRC patients. **A** The level of IL-22 in the serum of CRC patients and normal subjects. **B** The level of IL-22 in the tumor and adjacent non-tumor tissues of CRC patients. **P < 0.01 vs. normal or adjacent tissue group

Using ELISA, we found that IL-22 concentrations were markedly elevated in the serum of CRC patients compared to normal subjects (Fig. 1A). Additionally, IL-22 expression was also significantly increased in CRC tissues compared to adjacent non-tumor tissues (Fig. 1B). This findings suggested that IL-22 was hyperactivated in CRC tissues, potentially playing a role in tumor growth and metastasis.

### IL-22 promotes HCT116 cell proliferation

**Fig. 2:**
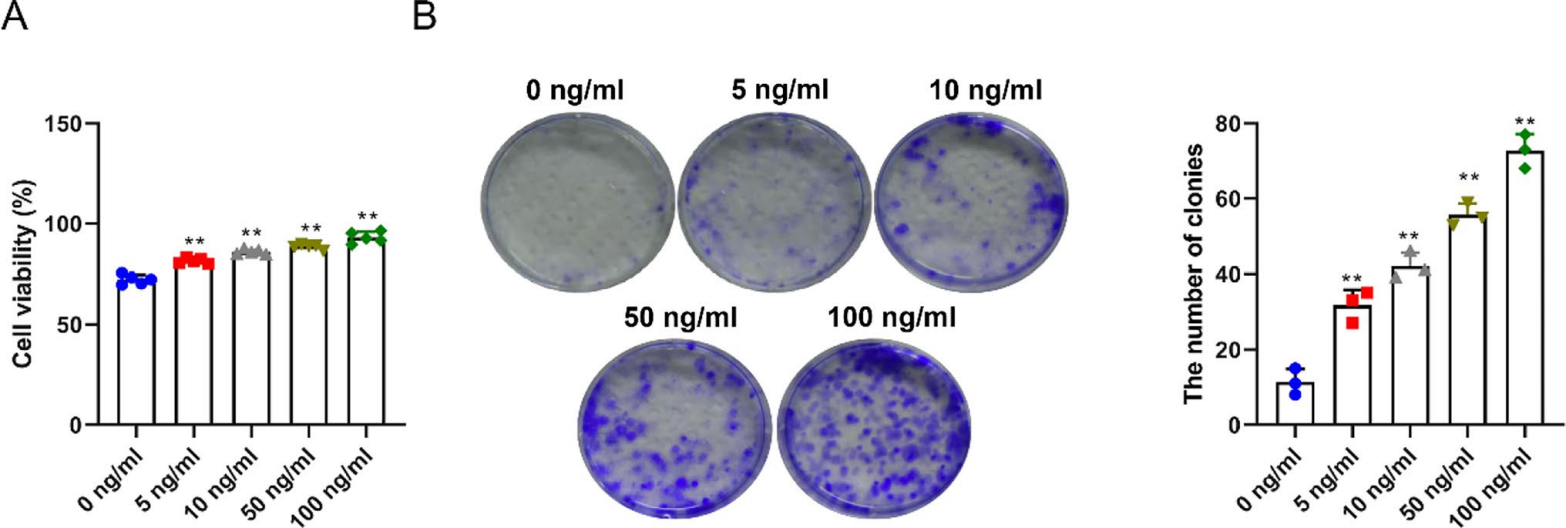
IL-22 promotes HCT116 cell proliferation. **A** Cell viability of HCT116 cells treated with different concentrations (0, 5, 10, 50, and 100 ng/mL) of IL-22 was assessed by CCK-8 assay. **B** The colony formation assay was used to assess the formation of cell colonies after treatment of HCT116 with different concentrations (0, 5, 10, 50, and 100 ng/mL) of IL-22. Data representation: Mean ± SD (*n* = 3). **P < 0.01 vs. the 0 ng/mL group

To assess the effect of IL-22 on CRC cell proliferation, we performed CCK-8 and colony formation assays on HCT116 cells. The CCK-8 assay demonstrated a significant concentration-dependent increase in cell viability with IL-22 treatment at concentrations of 5, 10, 50, and 100 ng/mL compared to the 0 ng/mL control (Fig. 2A). Similarly, The colony formation assay showed a notable increase in the number of colonies in these treatment groups, again in a concentration-dependent manner (Fig. 2B). These results indicated that, IL-22 significantly enhanced CRC cell viability and colony formation. For subsequent experiments, 100 ng/mL of IL-22 was selected based on the observed maximal increase in cell viability and colony formation at this concentration.

### IL-22 activates the PI3K/AKT pathway in HCT116 cells

**Fig. 3:**
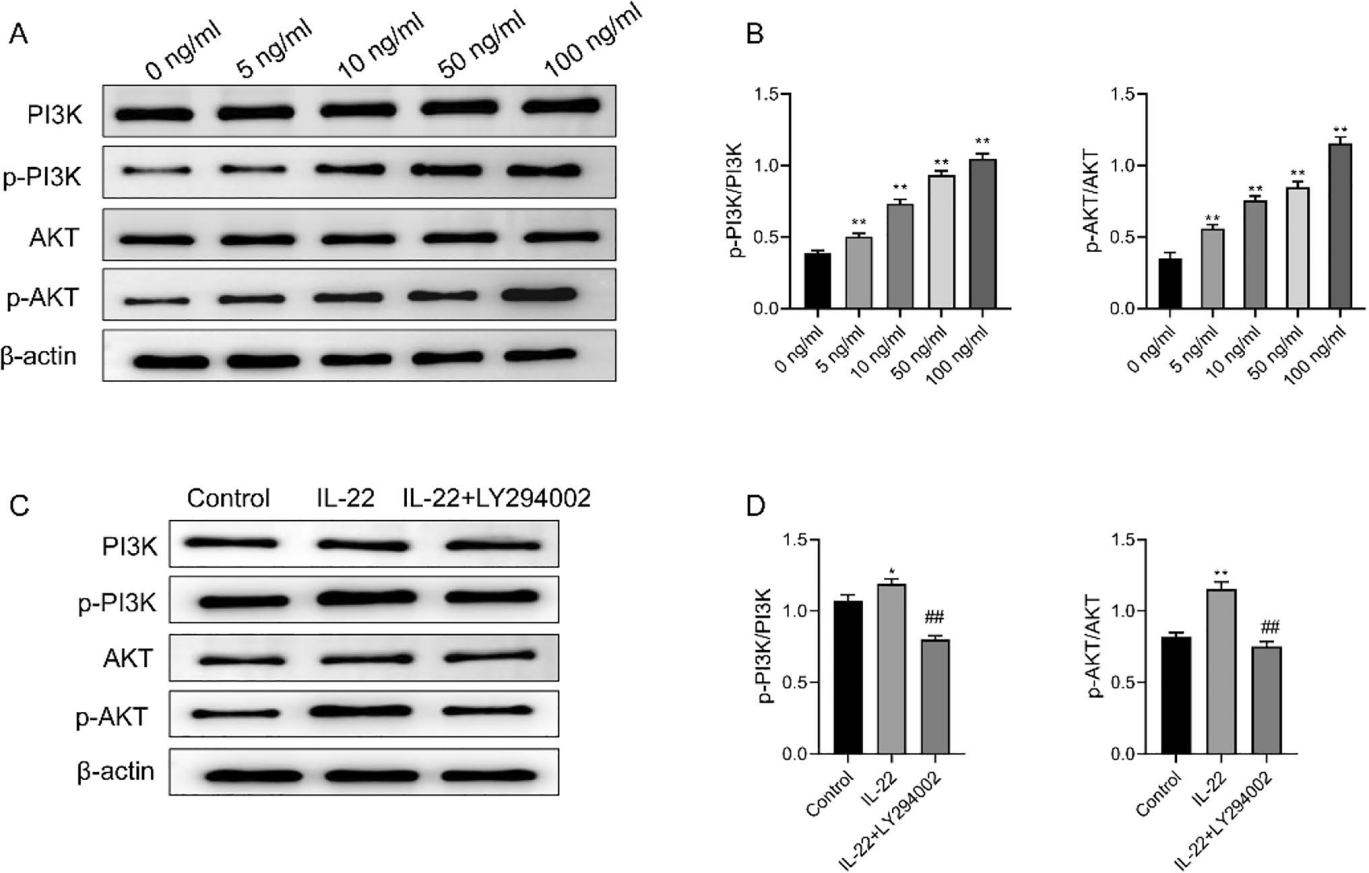
IL-22 activates the PI3K/AKT pathway in HCT116 cells. **A** The protein bands of PI3K, p-PI3K, AKT, and p-AKT in HCT116 cells treated with IL-22 at concentrations of 0, 5, 10, 50, and 100 ng/mL. **B** The relative expression of p-PI3K/PI3K and p-AKT/AKT. **C** The protein bands of PI3K, p-PI3K, AKT, and p-AKT in control, IL-22 (100 ng/mL), and IL-22 (100 ng/mL) + LY294002 groups. **D** The relative expression of p-PI3K/PI3K and p-AKT/AKT. Data representation: Mean ± SD (*n* = 3). **P < 0.01 vs. the 0 ng/mL or control group; ^##^*P* < 0.01 vs. the IL-22 group

To investigate the signaling pathways involved in IL-22-mediated effects, we examined the PI3K/AKT pathway. Western blot analysis revealed that IL-22 treatment led to a concentration-dependent increase in the levels of p-PI3K and p-AKT, without no significant effects on the total expression of PI3K and AKT (Fig. 3A, B). As demonstrated in Fig. 3C, D, treatment with IL-22 (100 ng/mL) alone remarkably increased p-PI3K and p-AKT levels, whereas the combined treatment with IL-22 and PI3K inhibitor (LY294002) resulted in a marked decrease in these phosphorylation levels, and the total expression of PI3K and AKT remained unchanged. These results indicated the regulatory function of IL-22 in the modulation of PI3K/AKT signaling pathway.

### Inhibition of the PI3K/AKT pathway attenuates the effects of IL-22 on HCT116 cells

**Fig. 4:**
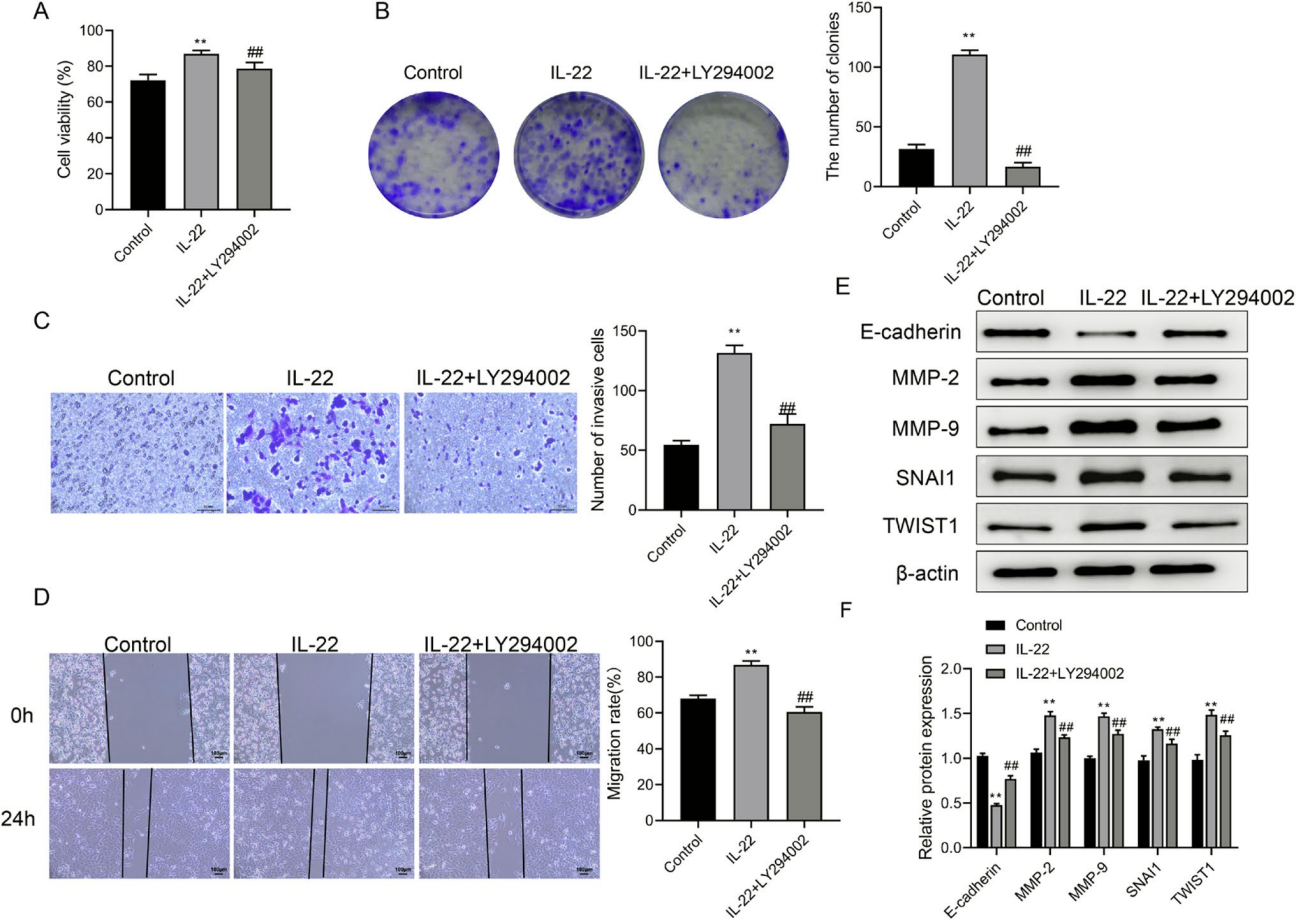
Inhibition of the PI3K/AKT pathway attenuates the effects of IL-22 on HCT116 cells. **A** Cell viability was assessed using CCK-8 assay in control, IL-22 (100 ng/mL), and IL-22 (100 ng/mL) + LY294002 groups. **B** Colony formation assay was performed to detect the number of cell colonies in control, IL-22 (100 ng/mL), and IL-22 (100 ng/mL) + LY294002 groups. **C** Transwell assay was performed to detect the cell invasion in control, IL-22 (100 ng/mL), and IL-22 (100 ng/mL) + LY294002 groups. Scale bar = 50 μm. **D** Scratch assay was performed to detect the cell migration in control, IL-22 (100 ng/mL), and IL-22 (100 ng/mL) + LY294002 groups. Scale bar = 100 μm. **E** The protein bands of E-cadherin, MMP-2, MMP-9, SNAI1 and TWIST1 in control, IL-22 (100 ng/mL), and IL-22 (100 ng/mL) + LY294002 groups. **E** The relative expression of E-cadherin, MMP-2, MMP-9, SNAI1 and TWIST1. Data representation: Mean ± SD (*n* = 3). **P < 0.01 vs. the control group; ^##^*P* < 0.01 vs. the IL-22 group

We next examined whether the effects of IL-22 on HCT116 cell proliferation and metastasis were mediated through the PI3K/AKT pathway. Compared to the control group, IL-22 treatment (100 ng/mL) caused marked increases in CRC cell viability and the number of cell colonies (Fig. 4A, B). However, these effects were significantly attenuated by the addition of LY294002, suggesting that IL-22 promotes cell growth and proliferation through the PI3K/AKT signaling.

Transwell and scratch assays demonstrated that IL-22 treatment significantly enhanced the invasion and migration capabilities of HCT116 cells, while the combined treatment with IL-22 and LY294002 inhibited these effects (Fig. 4C, D). In addition, Western blotting results showed that IL-22 treatment resulted in decreased E-cadherin levels and increased levels of MMP-2, MMP-9, SNAI1 and TWIST1 (Fig. 4E, F). By contrast, the addition of LY294002 effectively reversed these consequence. These observations indicated that IL-22 might promote cell migration and invasion by modulating these proteins through PI3K/AKT pathway.

## Discussion

According to the World Health Organization, CRC is the fourth most common and fatal cancer worldwide. Although CRC pathogenesis is not fully understood, cancer-related inflammation is an important component of CRC development [17–19]; it contributes to CRC progression by promoting cell proliferation, inhibiting apoptosis, inducing angiogenesis, and modulating immune responses [20, 21].

Over the past few years, researchers have extensively investigated the mechanisms of CRC development, particularly IL family molecules [22]. IL-22 is an immune- and inflammation-related cytokine that plays crucial regulatory roles in various pathological conditions [23, 24]. Baker et al. found increased IL-36 expression during CRC development and an association between decreased IL-36 receptor expression and improved patient outcomes [25]. Differential gene expression analysis results revealed novel roles for the IL-36/IL-17/IL-23 axis and validated their findings using clinical samples from patients and cell lines [25]. IL-36γ, together with IL-17α or IL-22, synergistically induces the expression of different genes involved in the IL-17/IL-23 axis and additively induces colon cancer cell proliferation [25], supporting a tumor-promoting role for inflammatory factors, such as IL-22 and IL-36, in CRC.

Cui et al. reported that most CRCs are caused by precancerous adenomas [26]. After exposure to multiple inflammatory microenvironmental factors, molecules such as IL-4, IL-6, IL-8, IL-17α, IL-22, IL-23, IL-33, and interferon-γ, induce complex genetic/molecular changes in precancerous stem cells (pCSC) to gradually form cancer stem cells (CSCs) [26]. The pCSC/CSC niche is an inflammation-dominated environment containing diverse cytokines that are critical communication factors between pCSCs/CSCs and their niche and play a decisive role in promoting CRC development, progression, and metastasis [26]. The above evidence demonstrates the importance of IL-22 in CRC. Thus, it is important to investigate the potential of IL-22-related therapy and understand the mechanism of CRC progression and its role in developing new treatment strategies.

Based on the above objectives, this study examined IL-22 expression in the serum and tissues of CRC patients. Serum levels of IL-22 were significantly higher in CRC patients than in normal subjects, suggesting that IL-22 may affect developing CRC and may be associated with disease progression and prognosis. Increased serum levels of IL-22 may reflect the degree of inflammation and production and release of IL-22 by tumor cells. Therefore, IL-22 is a potential inflammatory marker for early diagnosis and prognostic evaluation of CRC. Additionally, IL-22 levels were significantly higher in CRC tissues than adjacent noncancerous tissues, suggesting that IL-22 is abnormally expressed during CRC development and may be associated with tumor proliferation and invasion. Investigating IL-22 expression levels in CRC tissues provides a better understanding of the mechanisms of IL-22 during tumorigenesis and the association between IL-22 and CRC development. Moreover, studying IL-22 expression in CRC tissues offers valuable clues for further exploration of the IL-22 signaling pathway and its driving mechanism, and new therapeutic strategies can be expected. This study also assessed the effects of IL-22 on CRC cell proliferation viability using CCK-8 and cell colony formation assays. Results showed that IL-22 significantly increased the viability and number of colonies in a concentration-dependent manner, indicating that high IL-22 levels may be associated with CRC cell proliferation. Transwell and scratch assays showed a marked increase in CRC cell invasion and migration after IL-22 treatment, further supporting the important role of IL-22 in CRC progression.

The PI3K/AKT signaling pathway is an important intracellular signaling pathway that regulates various biological processes such as cell proliferation, survival, migration, metabolism, and apoptosis [27–29]. This pathway is involved in various diseases, including tumor development and progression. AKT phosphorylates multiple target proteins in cells, participating in various cellular physiological processes. Phosphorylation can promote cell survival and proliferation and inhibit apoptosis and autophagy [30, 31]. AKT can activate multiple cyclin-dependent kinases through phosphorylation and promote cell entry into cell division [30, 31]. In addition, AKT can phosphorylate and inhibit apoptosis-related proteins, such as Bad and caspase-9, to prevent apoptosis [30, 31]. AKT can also regulate cell survival and metabolism by phosphorylating forkhead box proteins of class O (FOXO) family transcription factors, inhibiting their nuclear translocation and transcriptional activity [30, 31].

Cassio et al. analyzed mutations in the main components of the PI3K/AKT pathway (*PI3KCA*, phosphatase and tensin homolog (*PTEN*), *PIK3R1*, and *PIK3R2*) and Wnt/β-catenin pathway (*APC*, *CTNNB1*, *AXIN1*, and *AXIN2*) in several open-access CRC clinical cohorts [32]. Interestingly, 104 (14.7%) of the 705 patients had *PIK3CA* mutations in the PI3K pathway, whereas 36 (5.1%) had *PIK3CA* and *PTEN* mutations. *PI3K* mutations were more frequent in the early stages of CRC progression (stages I and II) when comparing patients with no mutations and those with *PI3K* mutations alone (56.4% vs. 32.1%), suggesting that genetic variants in the AKT/PI3K pathway genes may be associated with early clinicopathological features in CRC patients. In other words, these findings support that the PI3K pathway plays a critical role in CRC pathogenesis and progression, providing new biomarkers for individualized treatment and prognostic evaluation and offering new directions for future therapeutic research and clinical practice.

Chen et al. considered CRC a disease with complex pathogenesis and easy metastasis and pointed out that its development involves abnormalities in various signaling pathways, such as PI3K/AKT, nuclear factor-κB, mitogen-activated protein kinase, Wnt/β-catenin, epidermal growth factor receptor, tumor protein 53, transforming growth factor-β, and immunoregulatory pathways [33]. These pathways affect the proliferation, apoptosis, cell cycle, migration, invasion, autophagy, epithelial-mesenchymal transition, angiogenesis, and chemoresistance of CRC cells. Excessive activation of AKT can promote tumor cell proliferation, survival, and invasion and inhibit apoptosis, indicating a close relationship between excessive activation of the PI3K/AKT signaling pathway and tumor development. Therefore, the PI3K/AKT signaling pathway has become a vital target for the study and treatment of tumors. Tumor growth and proliferation are suppressed by inhibiting PI3K or AKT, which interferes with the PI3K/AKT signaling pathway.

**Fig. 5:**
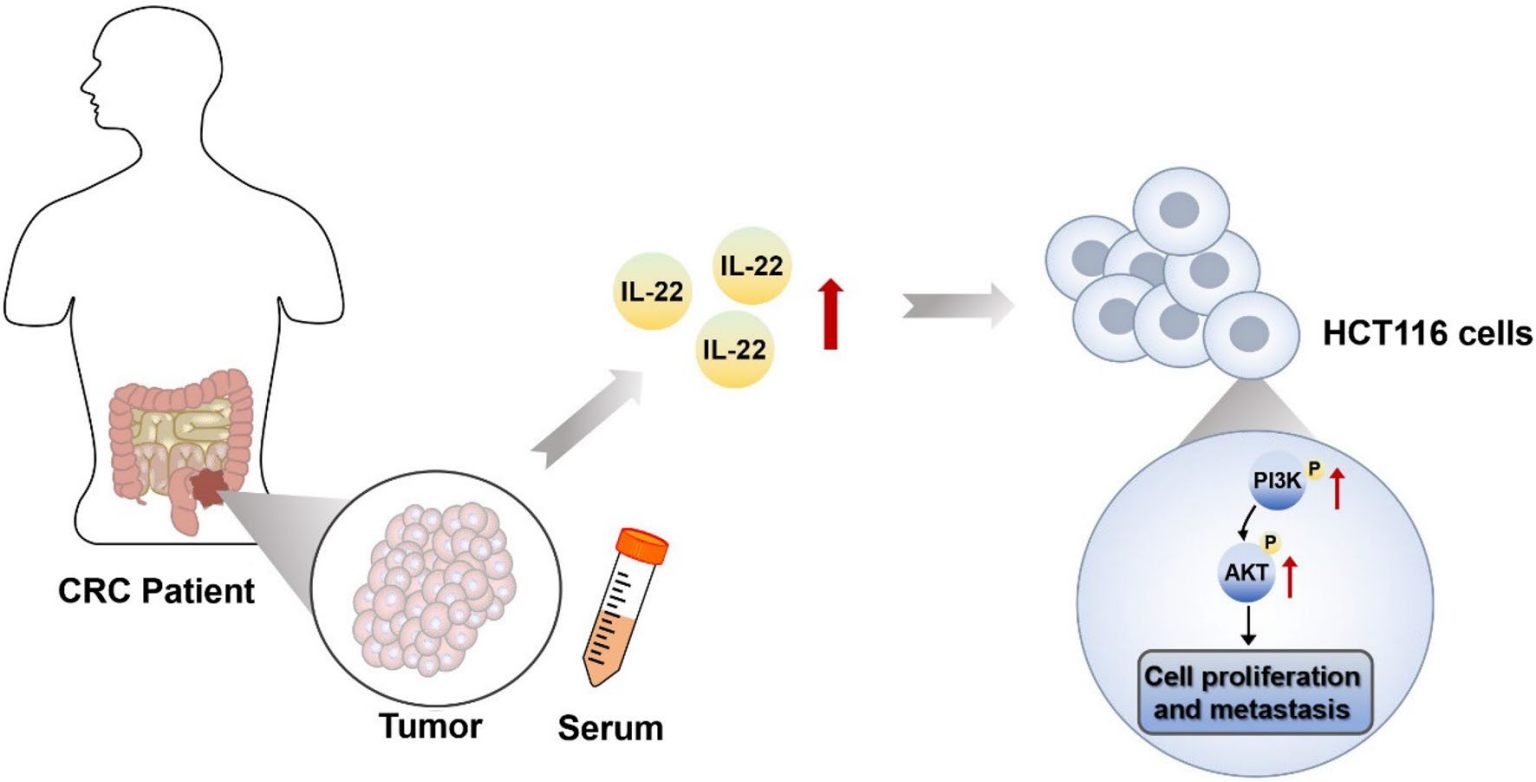
The diagram of the potential mechanism of IL-22 promoting the proliferation and metastasis in HCT116 cells

In this study, Western blotting analysis showed that IL-22 activated the PI3K/AKT signaling pathway and inhibited E-cadherin expression, suggesting that the PI3K/AKT signaling pathway may contribute to IL-22-induced cell phenotypic transition and that IL-22 may promote the epithelial-to-mesenchymal transition to enhance cell depolymerization and invasion. Meanwhile, the increase in MMP-2 MMP-9, SNAI1 and TWIST1 protein levels in the IL-22 group suggests that IL-22 may regulate MMP expression by activating the PI3K/AKT signaling pathway, enhancing tumor cell invasion and metastasis. Increased E-cadherin protein levels in the IL-22 + LY294002 group compared to the IL-22 group indicated that inhibition of PI3K/AKT signaling reversed IL-22-induced phenotypic shifts, promoted epithelial cell accumulation, and reduced invasion. At the same time, the decrease in MMP-2, MMP-9 SNAI1 and TWIST1 protein levels in IL-22 + LY294002 cells indicated that inhibition of the PI3K/AKT signaling pathway reduced MMP expression, attenuating tumor cell invasion and metastasis. Collectively, IL-22 may promote tumor cell invasion and metastasis by activating the PI3K/AKT signaling pathway, and inhibition of the PI3K/AKT signaling pathway may reverse the effects of IL-22 (Fig. 5).

Although this study thoroughly investigated the role of IL-22 in CRC, it has some limitations. Only in vitro experiments were performed, and the accuracy and feasibility of the findings require further validation in living models and clinical samples. The downstream mechanisms of IL-22 and interactions with other signaling pathways were not explored in-depth. In addition, the sample size was small; therefore, despite significant results, further investigation with a larger sample size is needed to improve the reliability of the statistics.

## Conclusion

In summary, this study demonstrated that IL-22 and PI3K/AKT signaling pathways play important regulatory roles in CRC development and revealed the mechanism of IL-22 in tumor cell invasion and metastasis. These findings help understand the role of IL-22 in cancer development, especially in tumor cell metastasis and invasion stages, and provide a theoretical basis for developing molecular therapeutic strategies targeting IL-22 and AKT signaling pathways.

### Supplementary Information

Supplementary Material 1.

Supplementary Material 2.

## Data Availability

Datasets used in this article are available from the corresponding author on reasonable request.
