# Supplementary material for: IL-22 activates the PI3K-AKT pathway to promote colorectal cancer cell proliferation and metastasis
Source: Discov Oncol. 2024 Jul 29;15:317. doi: 10.1007/s12672-024-01169-9 (PMC11286610; doi:10.1007/s12672-024-01169-9)
Supplement: Supplementary file 1 — Supplementary Material 1. [file 12672_2024_1169_MOESM1_ESM.pdf]

Figure 3A

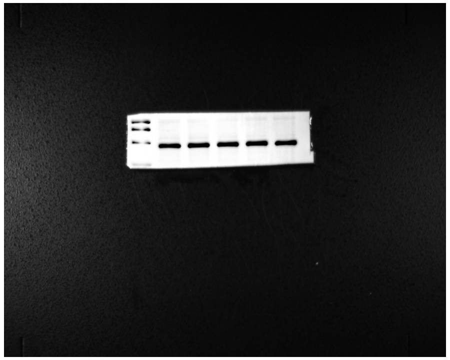

The original Western Blot images of PI3K in Figure 3A. From left to right: 0 ng/ml, 5 ng/ml, 10 ng/ml, 50 ng/ml, 100 ng/ml.

Figure 3A

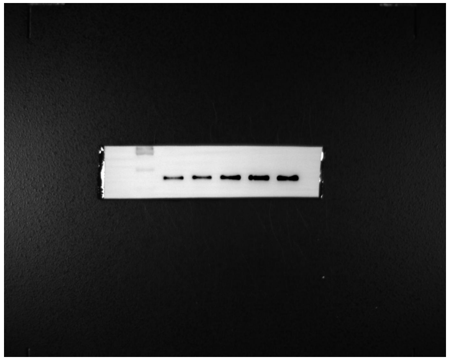

The original Western Blot images of P-PI3K in Figure 3A. From left to right: 0 ng/ml, 5 ng/ml, 10 ng/ml, 50 ng/ml, 100 ng/ml.

Figure 3A

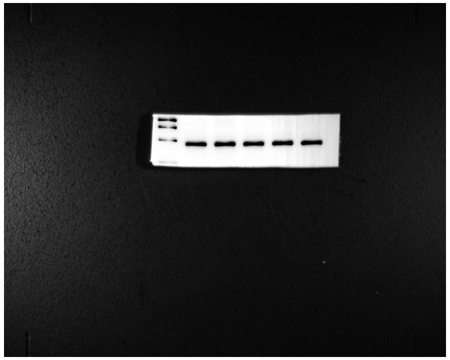

The original Western Blot images of AKT in Figure 3A. From left to right: 0 ng/ml, 5 ng/ml, 10 ng/ml, 50 ng/ml, 100 ng/ml.

Figure 3A

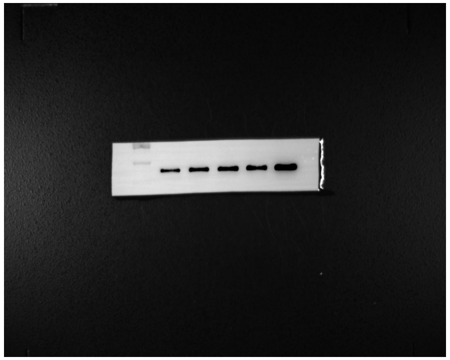

The original Western Blot images of P-AKT in Figure 3A. From left to right: 0 ng/ml, 5 ng/ml, 10 ng/ml, 50 ng/ml, 100 ng/ml.

Figure 3A

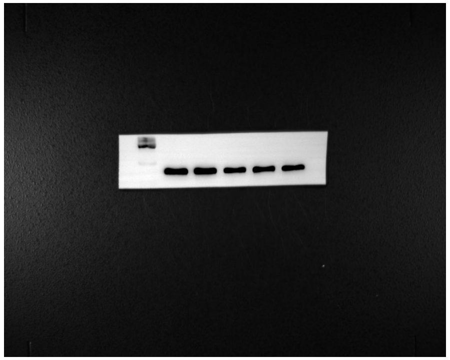

The original Western Blot images of β-actin in Figure 3A. From left to right: 0 ng/ml, 5 ng/ml, 10 ng/ml, 50 ng/ml, 100 ng/ml.

Figure 3C

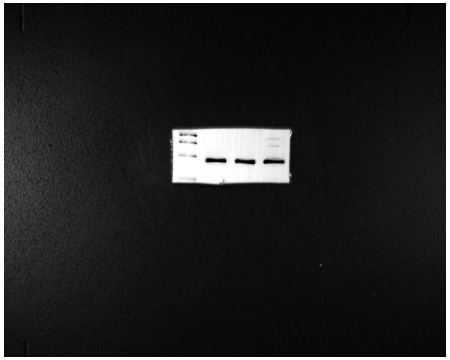

The original Western Blot images of PI3K in Figure 3C. From left to right: Control, IL-22, IL-22+LY294002.

Figure 3C

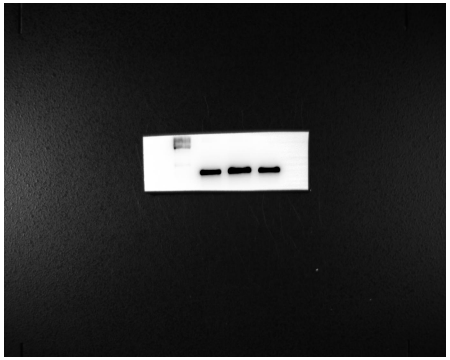

The original Western Blot images of P-PI3K in Figure 3C. From left to right: Control, IL-22, IL-22+LY294002.

Figure 3C

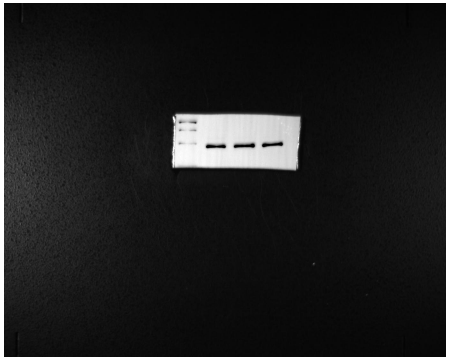

The original Western Blot images of AKT in Figure 3C. From left to right: Control, IL-22, IL-22+LY294002.

Figure 3C

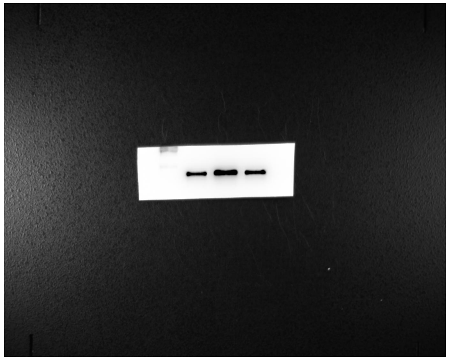

The original Western Blot images of P-AKT in Figure 3C. From left to right: Control, IL-22, IL-22+LY294002.

Figure 3C

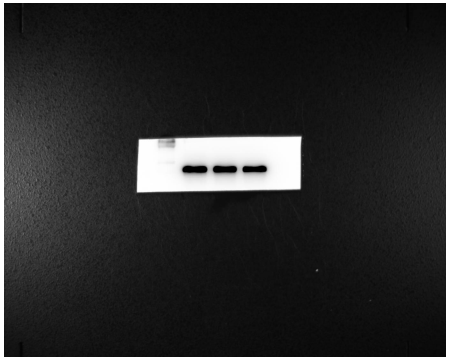

The original Western Blot images of β-actin in Figure 3C. From left to right: Control, IL-22, IL-22+LY294002.

Figure 4E

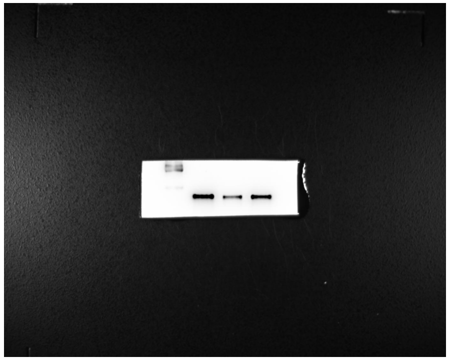

The original Western Blot images of E-cadherin in Figure 4E. From left to right: Control, IL-22, IL-22+LY294002.

Figure 4E

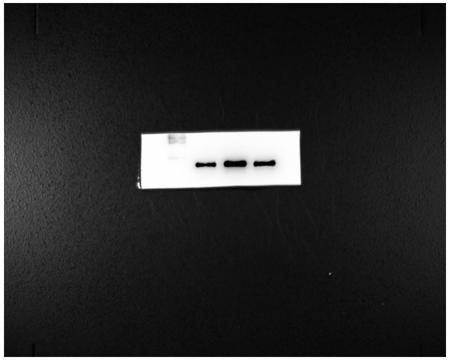

The original Western Blot images of MMP-2 in Figure 4E. From left to right: Control, IL-22, IL-22+LY294002.

Figure 4E

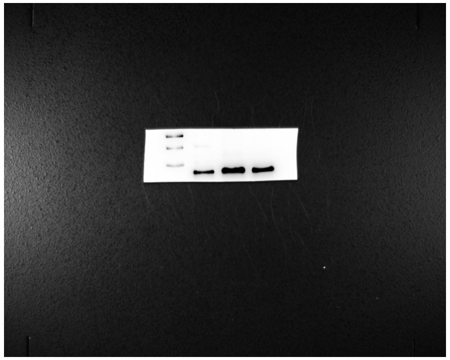

The original Western Blot images of MMP-9 in Figure 4E. From left to right: Control, IL-22, IL-22+LY294002.

Figure 4E

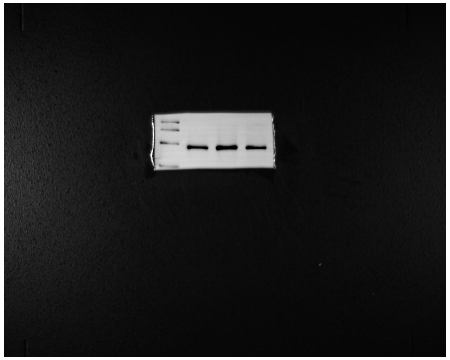

The original Western Blot images of SNAI1 in Figure 4E. From left to right: Control, IL-22, IL-22+LY294002.

Figure 4E

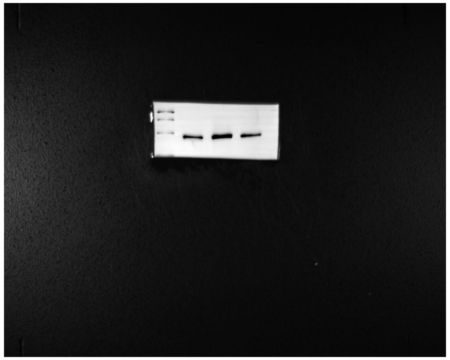

The original Western Blot images of TWIST1 in Figure 4E. From left to right: Control, IL-22, IL-22+LY294002.

Figure 4E

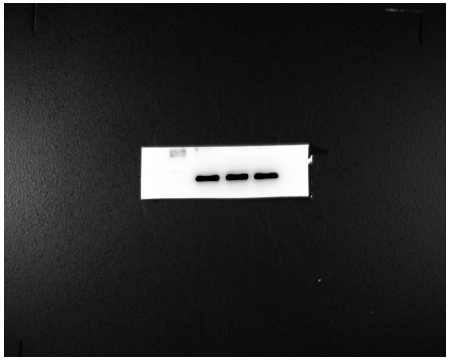

The original Western Blot images of β-actin in Figure 4E. From left to right: Control, IL-22, IL-22+LY294002.
