# Supplementary material for: IL-22 activates the PI3K-AKT pathway to promote colorectal cancer cell proliferation and metastasis
Source: Discov Oncol. 2024 Jul 29;15:317. doi: 10.1007/s12672-024-01169-9 (PMC11286610; doi:10.1007/s12672-024-01169-9)
Supplement: Supplementary file 2 — Supplementary Material 2. [file 12672_2024_1169_MOESM2_ESM.docx]

Supplementary Table 1 Clinical information of the patient.

| Characteristics |  | Patients (n = 66) |
| --- | --- | --- |
| Age |  | 61.18±12.52 |
| Sex | Female | 27 (40.91%) |
|  | Male | 39 (59.09%) |
| Current smoking |  | 22 (33.33%) |
| Current drinking |  | 15 (22.73%) |
| History of surgery |  | 20 (30.30%) |
| Diabetes |  | 7 (10.61%) |
| Hypertension |  | 24 (36.36%) |
| Coronary heart disease |  | 4 (6.06%) |
| Nutritional status |  |  |
|  | Medium | 61 (92.42%) |
|  | Good | 5 (7.58%) |
| BMI (kg/m^2^) |  | 24.51±3.88 |
| Hemoglob (g/L) |  | 120.86±22.78 |
| Albumin (g/L) |  | 40.33±4.21 |
| Interval between last dose of laxatives and colonoscopy (h) |  | 0.59±0.50 |

Data are mean ± SD or n (%). BMI: Body mass index.
